# A Flexible Strain Sensor Based on Embedded Ionic Liquid

**DOI:** 10.3390/s21175760

**Published:** 2021-08-26

**Authors:** Huiyang Zhang, Andrew Lowe, Anubha Kalra, Yang Yu

**Affiliations:** Institute of Biomedical Technologies, Auckland University of Technology, Auckland 1010, New Zealand; andrew.lowe@aut.ac.nz (A.L.); anubha.kalra@aut.ac.nz (A.K.); yang.yu@aut.ac.nz (Y.Y.)

**Keywords:** strain sensor, flexible sensor, motion detection, wearable device, ionic liquid, fluid channel

## Abstract

We present a simple-structured strain sensor based on a low-cost ionic liquid. The ionic liquid was made of sodium chloride/propylene glycol solution and was embedded in a linear microfluidic channel fabricated using Ecoflex. The proposed sensor is capable of measuring strain up to 100% with excellent repeatability. The highest gauge factor is obtained as 6.19 under direct current excitation and 3.40 under alternating current excitation at 1 kHz. The sensor shows negligible hysteresis and overshoot, and survived 10,000 rapid stretch-release cycles of a 100% peak strain with a minor deviation in the response signal. The sensor can be mounted to different locations on the human body and suits a variety of applications in the field of motion detection, human–machine interface and healthcare monitoring.

## 1. Introduction

Due to the emerging trend in wearable technologies, flexible strain sensors that can withstand large deformation have received a considerable amount of interest in recent years. Compared with conventional strain gauges made of metal or semiconductive material, polymer-based strain sensors exhibit significantly higher mechanical flexibility, making them favorable candidates for a wide range of wearable applications such as human–machine interfaces [1,2], motion detection systems [3,4], soft robotics [5,6], and healthcare monitoring devices [7,8]. Currently, flexible strain sensors are developed using various approaches. A large number of sensors are based on crack propagation of a thin layer of conductive particles such as carbon nanotubes (CNTs) [9,10,11], graphene [12,13] and metal nanowires [14,15,16], coated on a polymer substrate. Alternatively, the mechanism of disconnection in percolation networks is also utilized in many sensors, where nanoparticles with high aspect ratios, typically CNTs [17,18,19], are uniformly dispersed into a polymer matrix to form numerous percolation networks [20] which partially break upon stretching. In addition, capacitive strain sensors have been reported with a sandwich structure which consists of two electrode sheets made of conductive nanoparticles [4,21,22] and a thin dielectric layer in-between. Although flexible strain sensors have superior stretchability in comparison with conventional metallic strain sensors, they have several drawbacks that limit their utility in practice. Previous research shows that piezoresistive sensors based on microcracks or percolation networks [23,24,25] have issues with large hysteresis and overshoot due to the viscoelastic property of the polymer matrix, which causes a delay in the electrical response of its incorporated conductive surfaces and networks [26,27,28]. Capacitive sensors demonstrate high signal linearity and low hysteresis [4,21,22]; however, they have low capacitance values which are typically in the range from 2.2 pF to 35 pF [21,29,30] at zero strain and are further lowered as the strain increases. Measurement of capacitance at this scale is challenging and usually requires an LCR meter or a capacitance meter with a high precision, which is not feasible for wearable applications.

Recently, strain sensors embedding liquid sensing element have been introduced using various types of fluids such as room-temperature liquid metal [31], electrolytes gels [32] and ionic liquid [33,34,35,36]. These liquid-based sensors successfully avoided the polymer relaxation issue and the low signal reading issue possessed by aforementioned solid-based sensors. However, their effective use is limited due to the high cost and surface tension of liquid metal and the poor electro-mechanical stability of electrolytes gels [37]. Moreover, the synthesis of electrolytes gels usually requires the participation of highly toxic compounds such as frequently used acrylamide (AM), $N,N^{\prime }$-methylene-bis-acrylamide (MBAA) and ammonium persulfate (APS) [32,38,39,40], which often leave residual in the final product. In addition, sensors based on ionic liquid often involves sophisticated design for their channel morphology [31,33,41] and therefore require complicated fabrication procedures.

Herein, a simple-structured strain sensor based on a low-cost and nontoxic liquid sensing medium is proposed. The sensor utilizes a solution of sodium chloride (NaCl) dissolved in propylene glycol (PG) ($C_{3}H_{8}O_{2}$) as its strain sensing element. A linear channel for embedding the NaCl/PG solution was fabricated by casting Ecoflex into a 3D printed mold. The sensor was then tested under various conditions to examine its characteristics and strain sensing performance. The sensor is capable of measuring strain of up to 100% and has a Young’s modulus of 15.92 kPa, which is similar to that of human skin. The signal of the sensor was found to have reliance on the excitation frequency and the ambient temperature variation. Notably, with a linear channel structure fabricated using a simple method, this sensor possesses negligible hysteresis and overshoot behaviour for both cyclic and dynamic strain. A durability test which involves the sensor experiencing 10,000 cycles of loading and unloading was performed to validate the sensor supports the long-term operation. Moreover, the proposed sensor was mounted to various locations on the human body to detect motions and monitor breathing cycles.

## 2. Experiment

### 2.1. Characterization of Ionic Liquid

**Figure 1:**
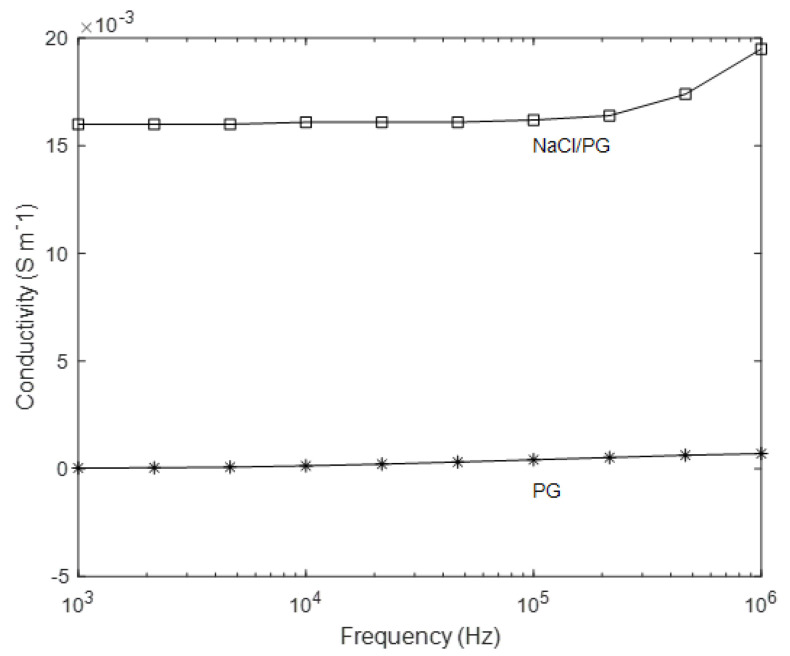
Electrical conductivity of pure PG and NaCl/PG solution in the frequency range between 1 kHz and 1 MHz.

The strain-sensing ionic liquid is prepared by dissolving sodium chloride (NaCl) (ACS reagent, ≥99.0%, Sigma-Aldrich, Auckland, NZ) in propylene glycol (PG) (USP grade, Pure Nature Ltd, Auckland, NZ). NaCl is commonly known as salt; it dissociates into charge-carrying sodium cations and chloride anions when dissolved in PG, a viscous and colorless liquid solvent. PG has the lowest toxicity amongst all the glycols and has extensive usage in food additives, antifreeze, hydraulic fluid, and pharmaceutical solvents for cosmetics [42]. Unlike water, which can reduce into oxygen and hydrogen bubbles when electrically charged under Direct Current (DC) [43] and block the liquid channel, PG does not form gas bubbles and therefore is more suitable for constituting the strain-sensing liquid. The solution was made at a concentration of 5 g of NaCl per liter of PG. The dissociation of NaCl into ions was accelerated by using a ultrasonicator (VC-505, John-Morris Scientific, Sydney, Australia) set at 20 kHz frequency and 40% amplitude. The obtained solution was degassed using a vacuum chamber to remove the air bubbles. The electrical conductivity of liquid was measured with MFIA Impedance Analyzer (Zurich Instruments AG, Zurich, Switzerland), under a sweep frequency from 1 kHz to 1 MHz. As shown in Figure 1, in the low-frequency range, the electrical conductivity of pure PG was measured to be (3.59 × 10${}^{-5}$) S/m, and the conductivity of the proposed NaCl/PG solution was measured to be (1.60 × 10${}^{-2}$) S/m.

### 2.2. Fabrication of the Strain Sensor

**Figure 2:**
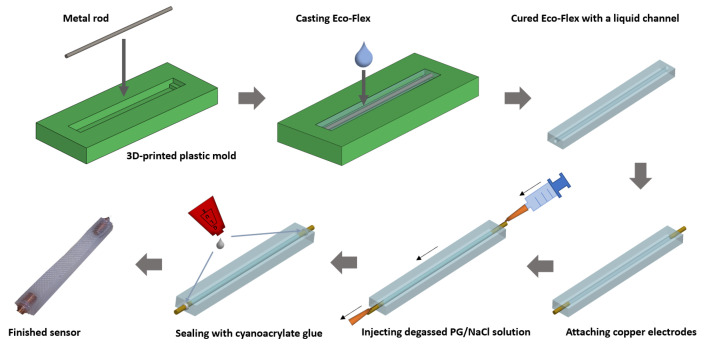
The fabrication procedure of the flexible strain sensor.

The fabrication process of the strain sensor is illustrated in Figure 2. A plastic mold with a cuboid-shaped cavity was fabricated using the 3D printing technique. A metal rod with 1 mm diameter was placed onto elevated segments located at both ends of the cavity. To construct a stretchable channel for embedding a sensing liquid, part A and part B of silicone rubber (Eco-Flex 00-20, Smooth-on) were mixed at a mass ratio of 1:1 and thoroughly stirred. After degassing using a vacuum chamber to remove the enclosed air bubbles, the liquid mixture was slowly cast into a mold and cured for four hours at room temperature. Subsequently, the cured silicone rubber was released from its mold, along with the metal rod carefully extracted and left the silicone rubber with an empty channel in place. Two copper rods were attached to both ends of the channel to serve as electrodes. Then the degassed NaCl/PG solution was gently injected through the edge between the channel and electrode at one end using a syringe, while the air inside the channel was pushed out through a syringe needle at the other end till the channel is entirely filled with the liquid. In the end, the edges along the perimeters of the electrodes and the fluid channel were sealed using cyanoacrylate glue. The obtained sensor has a 10 mm by 5.4 mm dimension for the transverse cross section and a length of 40 mm for the liquid channel.

### 2.3. Characterization of Sensor Performance

**Figure 3:**
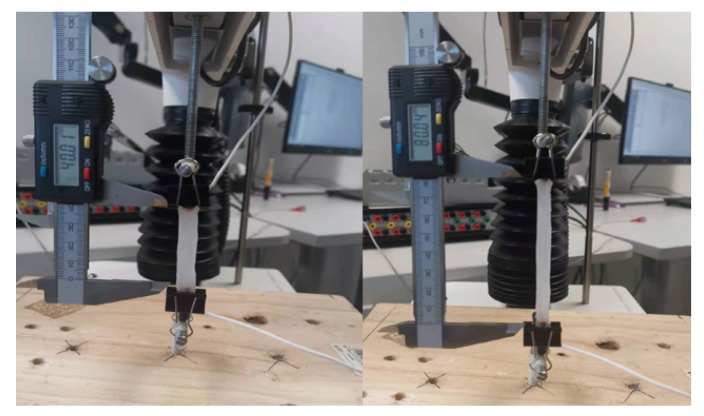
Sensor secured to the TA.XT-plus using conventional metal clips. **left**: unloaded state. **Right**: fully loaded state at 100% strain.

The strain-resistance response of the prepared strain sensor was evaluated using TA.XT-plus Texture Analyser (Stable Micro Systems Ltd, Godalming, UK) and MFIA Impedance Analyzer (Zurich Instruments AG, Zurich, Switzerland). The mechanical strain applied to the sensor is controlled by the TA.XT-plus using its provided Exponent software interface. The device offers a high motion control resolution of 0.001 mm and a wide range of speed control between 0.01 and 40 mm/s. The two ends of the sensor were mounted to the probes of TA.XT-plus using two simple metal clips which held the sensor firmly in place, as indicated in Figure 3. The sensor was connected to the MFIA through copper wires soldered to the electrodes at both ends of the sensor. The real part of the impedance of the prepared sensor was recorded using the LabOne software interface. The signal voltage of MFIA was set to 300 mV and the signal frequency was set to 1 KHz.

To comprehensively evaluate the effectiveness and the reliability of the prepared sensor, a range of parameters associated with sensor performance were tested. First, a mechanical tensile test for up to 100% strain was conducted to determine the mechanical characteristics of the sensor. Then the effect of excitation frequency was studied by recording the strain-resistance response of the sensor under several selected frequencies. The effect of ambient temperature on the resistance of the sensor was also investigated by placing the sensor into a water bath which is heated using a hot plate, while its resistance variance over a range of temperature was recorded. A cyclic loading test was performed in a stretch-release manner with different magnitudes of strain to verify the consistency in each measurement. In the meantime, the hysteresis of the sensor during stretch and release cycles was assessed. An overshoot test was performed by a repetitive stretch-and-hold experiment, in which the sensor was repetitively stretched to 100% strain at a strain rate of 100%/s (40 mm/s) and held for 5 s, before it was released to zero strain at the same strain rate. In addition, a dynamic loading test was conducted to examine the response of the sensor to the strain of incoherently selected magnitudes. Furthermore, a durability test was performed to ascertain the signal stability of the sensor as it undertook 10,000 cycles of 100% strain. Apart from the overshoot test and the durability test, the strain rates used in other tests were set to 10 mm/s by default. At the end, the sensor was attached to various spots on the human body, including a finger, a forearm, a thigh and the chest, to demonstrate its functions of detecting motions and monitoring breathing cycles.

## 3. Results and Discussion

### 3.1. Sensor Characteristics

**Figure 4:**
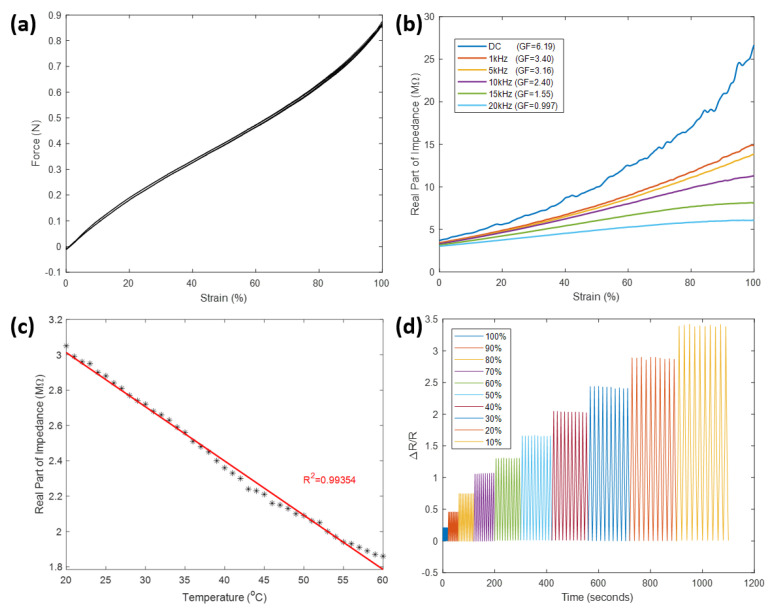
Characteristics of the strain sensor. (**a**): Force-strain relation determined by the mechanical tensile test. (**b**): Sensitivity variation due to the effect of the excitation frequency. (**c**): Resistance variation due to the effect of ambient temperature. (**d**): Relative change in resistance in the cyclic loading test.

**Mechanical tensile test** Elasticity is an important attribute for a wearable strain sensor. A sensor mounted to the human body should provide comfort and avoid limiting user movement. The relationship between the applied force and the strain applied to the prepared sensor was recorded by TA.XT-plus and plotted in Figure 4a. It can be seen stretching the sensor to strain at 100% only takes less than 1 N of force. The elasticity of a strain sensor is quantified by Young’s modulus. In tensile testing, Young’s modulus (*E*) is determined by dividing the stress (σ) by the applied strain (ϵ):

(1) $$E=\frac{\sigma }{\epsilon }$$

where the stress is calculated as the force per unit cross-sectional area. The Young’s modulus of the sensor was calculated to be 15.92 kPa, which is appreciably low in comparison to Young’s modulus of human skin that lies within a range between 5 kPa and 140 MPa [44]. When the sensor is directly attached to human skin, stretching the skin along with the sensor is less likely to cause an apparent sensation of skin tightening, indicating the sensor can offer good user comfort.

**Effect of excitation frequency** The excitation frequency of signal current has an impact on the electrical property of PG/water solution. The real part of impedance, or equivalently the resistance, of the solution was observed to decrease with increased excitation frequency. The relation between resistance and strain under different excitation frequencies is demonstrated in Figure 4b. It is apparent that an increase in the frequency not only reduced the resistance but also resulted in a decreased gauge factor (*k*). The term gauge factor is defined as the ratio of relative change in resistance to the applied strain (ϵ):

(2) $$k=\frac{\Delta R/R_{0}}{\epsilon }$$

The gauge factor was at the highest (GF = 6.19) when the resistance of the sensor was measured under DC, and it rapidly dropped below 1 under 20 KHz. Although the best sensitivity was present when DC was used, it was suggested that using DC may cause polarization of electrodes due to the charge separation problem [34], which results in unstable resistance measurement and hinders the sensor accuracy. To prevent this, 1 kHz was selected as the excitation frequency for the subsequent experiments. The gauge factor under this frequency is 3.40.

**Effect of ambient temperature** As an important organ for thermoregulation, human skin has an inconstant temperature [45], which may, together with the surrounding environment, affect the electrical property of the strain sensor attached to it. The effect of ambient temperature on the resistance of the proposed sensor was verified using a water bath method. Because the sensor is waterproof, it was directly submerged into deionized water contained in a beaker. The water was slowly heated up using a hot plate while its temperature was continuously measured using a standard thermometer. A resistance value is recorded every time when the temperature reading reaches an integer value between 20 °C and 60 °C. As illustrated in Figure 4c, the relationship between the resistance of the sensor and the ambient water temperature is approximately linear ($R^{2}=0.99$). Within the range of room temperature, the resistance of the sensor had a considerably small deviation which dropped from 3.05 MΩ at 20 °C to 2.88 MΩ at 25 °C. On the full scale, the resistance dropped to 1.86 MΩ when the water was heated to 60 °C. This suggests that even though the performance of the sensor is stable for general applications, the effect of temperature must be considered when the sensor is used under extreme conditions.

**Cyclic loading test** A reliable sensor should maintain a high level of consistency in its measured signal when strains of the same magnitude are repetitively applied. This consistency was verified by the cyclic loading test. Figure 4d shows the relative change in resistance of the prepared sensor as it was experiencing 10 stretch-release cycles at each strain with different magnitudes. It can be observed nearly identical results were obtained for all ten stretch-release cycles at each level of the applied strain from 10% to 100%, indicating excellent reproducibility of the sensor for cyclic strain measurement.

### 3.2. Evaluation of Strain Sensing Performance

**Figure 5:**
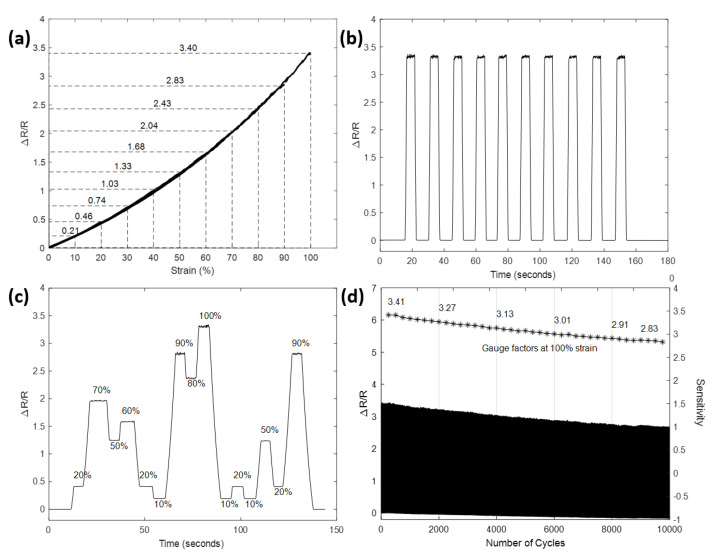
Evaluation of strain-sensing performance. (**a**): Signals corresponding to multiple stress-release cycles of different applied strain demonstrating negligible hysteresis. (**b**): Signals corresponding to ten rapid stretch-hold-release cycles of 100% peak strain demonstrating negligible overshoot. (**c**): Signals in response to dynamically applied strain. (**d**): Relative change in resistance and corresponding gauge factors over 10,000 stretch-release cycles.

**Hysteresis** A reliable sensor should output the same readings of resistance values when strains of the same magnitude is applied, despite how this strain is reached: either by stretching or releasing. As a matter of fact, however, this is often not well accomplished. For example, sensors based on the crack-propagation mechanism [46,47] and the network-disconnection mechanism [19,25] generally suffer from hysteresis to some extent, which results in a mismatch in their signal between loading and unloading. Hysteresis present in these sensors is mainly caused by the viscoelastic nature of their polymer matrix [4]. The hysteresis behavior of sensors can severely limit their measurement accuracy, particularly when their applications involve dynamically applied strain. The hysteresis behavior of the prepared sensor is demonstrated in Figure 5a, which indicates the relative change in resistance of the sensor during each stretch-release cycle, as each level of strain is successively applied and released, i.e., the sensor was first gradually stretched to 10% before it was released to the origin with the same speed; then a strain of 20% was applied and the same procedure was repeated for increasing the strain up to 100%. Notably, the measured signal shows an almost identical path of recovery to the path of stretching for all the tested strains. The curve of the signal for each level of strain also shows great consistency in their shared portion. Effectively, these curves in the plot are adequately close to being viewed together as a single curve, whose stretch path and release path are nearly indistinguishable, indicating the sensor is free of hysteresis and can work competently to measure dynamically changing strain within the tested range.

**Overshoot and dynamic stability** Overshoot of a strain sensor is a phenomenon that occurs once a stretching or releasing process is finished. The measurement signal continues its response to the already terminated mechanical stimuli for an extended period of time, before it finally draws back to the correct value. Like hysteresis, overshoot is also caused by the relaxation of the viscoelastic nature of polymers and is widely present in previous works [48,49,50]. When strain is suddenly reversed at the end of a stretching, polymers tend to release their stress instantly by mechanical deformations [51]. The strain rate at which a sensor is stretched or released has a substantial impact on the severity of its overshoot behavior. To examine the overshoot character of the prepared sensor, the sensor was stretched to 100% strain and held for 5 s before releasing it to zero strain. Due to the overshoot behavior is more obvious when a higher strain rate is applied [52], a high strain rate of 40 mm/s, which is equivalent to 100%/s, was selected here for both stretching and releasing the sensor. The relative change in resistance of the sensor during 10 cycles of repetition is shown in Figure 5b. It can be observed that there was no visible overshoot throughout each pause after the sensor reaches the maximum strain. This signifies that the prepared sensor can be used to accurately predict strain, even when the sensor deforms quickly at such a high strain rate. The sensor was further tested for its reliability under a dynamic loading environment by applying a set of strains with randomly selected magnitudes. As depicted in Figure 5c, there was no overshoot observed in the test and the measurement results were well matched with each other when the applied strain reached the same magnitudes (at 10%, 20% 50% and 90%). It is also worth noting that the values of relative change in resistance obtained in this test are in a close match with the one obtained in cyclic loading test aforementioned in Figure 5a, implying the sensor can function efficiently under both cyclic and dynamic loading conditions.

**Long-term durability** With the aim to assess the durability and the signal stability of the prepared strain sensor over long-term operations, the sensor was tested for its relative change in resistance over 10,000 cycles of stretch and release, with a peak strain of 100%. The strain rate was set to 100%/s for both stretching and releasing. The measured result over the 10,000 cycles is demonstrated in Figure 5d. The resistance of the sensor maintained a base value with a small deviation, and a peak value that slowly decreased as the sensor experienced more cycles. The sensitivity of the sensor was reduced by less than 10% after 5000 cycles and 18% after 10,000 cycles. However, it is worth noting that the sensitivity of the sensor was found recoverable to its initial value at about 3.40 after the sensor was left resting for several minutes. This phenomenon indicates the sensitivity variation of the sensor may be caused by the imbalanced ion distribution which gradually deteriorated during the continuous loading and unloading cycles, and the balance was restored after settling the sensor for a period of time. Overall, these experimental results confirm the prepared strain sensor has great durability over long term operation, as well as the capability of continuous measurement of large and rapid strain.

### 3.3. Applications

**Figure 6:**
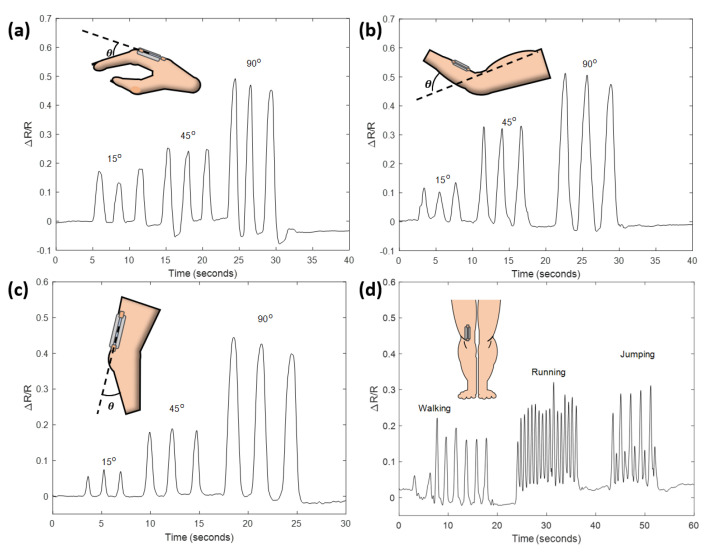
Relative change in resistance in response to different types of motions. (**a**): Finger bending. (**b**): Forearm bending. (**c**): Leg bending. (**d**): Signals corresponding to walking, running and jumping.

**Motion detection** To demonstrate the capability of the sensor as an effective wearable device for detecting various types of motions, the sensor was mounted to different spots on the human body, including fingers, forearms and legs, as illustrated in Figure 6a–c. The two ends of the sensor were attached to the indicated locations using duct tapes. The relative change in resistance of the sensor was recorded when each joint was bent to approximately 15, 45 and 90 degrees three times, respectively. The result obtained from each position exhibits excellent reproducibility, implying the sensor can function reliably at multiple locations and has the potential to be derived into a full-body motion detection system. The sensor mounted to the leg was further tested for its ability of discriminating ordinary actions such as walking, running and jumping. It can be seen from Figure 6d that these motions can be clearly distinguished from the acquired signal. The signal for walking shows steady rise-and-fall cycles which are corresponding to each step; while the signal for running has more frequent oscillation, indicating faster paces. The signal for jumping has a unique recoil at the end of every cycle due to the buffer to the impact upon each landing.

**Figure 7:**
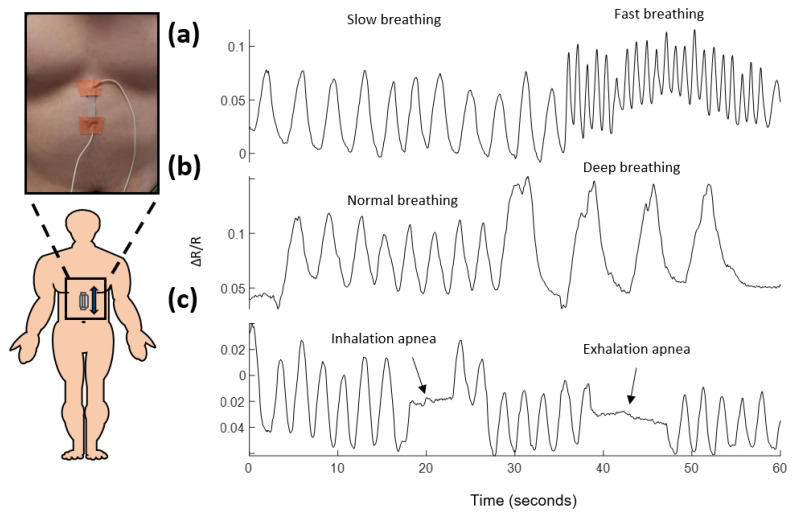
Relative change in resistance in response to different types of breathing events. (**a**): Breathing rate monitoring. (**b**): Breathing volume monitoring. (**c**): Apnea detection.

**Respiration monitoring** Furthermore, we demonstrate the employment of the prepared sensor for breath monitoring, including measurement of respiration rates, respiratory volumes and detection of apnea. The sensor was directly attached with a tape to the lower chest, in the region next to the diaphragm which separates the chest from the abdomen. The influence of lung ventilation during each breathing cycle on the epidermal strain variation is extreme at this location. The response of the sensor to the subtle skin deformation induced by different breathing conditions over a period of 60 s is illustrated in Figure 7a–c, respectively. As shown in Figure 7a, the sensor experienced deformation in sync with each breathing cycle during both slow breathing and fast breathing, and the frequency of the oscillation in its signal can be clearly used to measure the respiration rate. Figure 7b shows the response of the sensor to normal breathing and deep breathing. Apart from having a lower frequency, the portion of the signal corresponding to deep breathing also has a higher magnitude than the portion of normal breathing, indicating deep breathing has a lower respiration rate and larger respiratory volume. Apnea is the paused breathing that occurs typically during sleeping, and it may happen hundreds of times every night. Apnea is caused by involuntary overrelaxation of the muscles in the upper throat which blocks the airway. Prolonged apnea results in severe lack of oxygen which may cause brain damage and even death in the worst scenario. A demonstration of using the proposed sensor for detecting apnea is shown in Figure 7c. The interruption in the measured signal due to apnea during both inhalation and exhalation process were distinctively observed as two regions of flat signal that indicate temporary halt of breathing, implying the sensor as an effective tool for apnea detection.

## 4. Conclusions

In summary, a highly flexible strain sensor was accomplished by embedding NaCl/PG ionic liquid into a linear Ecoflex channel. The sensor possesses skin-alike elasticity with Young’s modulus of 15.92 kPa, good sensitivity with a gauge factor of 6.19 under DC excitation and 3.40 under AC excitation at 1 kHz, and high durability for surviving 10,000 cyclic loadings. Compared to previously reported sensors, the proposed sensor offers a simple structure and a low-cost sensing element while maintaining excellent attributes for sensing strain. These attributes include high consistency of signal in response to both cyclic and dynamic strain, negligible hysteresis, and no overshoot even under a strain rate as high as 100%/s. In addition, the sensor demonstrated its capability for detecting human motions at various parts of human body, and successfully performed for respiration monitoring. These results suggest that the proposed sensor is an effective tool for accurate strain sensing, and particularly useful for wearable applications in the field of motion detection systems, human–machine interfaces and healthcare monitoring devices.
